# The impact of lymph node density on survival of cervical cancer patients

**DOI:** 10.1038/sj.bjc.6605801

**Published:** 2010-07-13

**Authors:** S Polterauer, L Hefler, V Seebacher, J Rahhal, C Tempfer, R Horvat, A Reinthaller, C Grimm

**Affiliations:** 1 Department of Gynecology and Gynecologic Oncology, Medical University of Vienna, Waehringer Guertel 18-20,Vienna A-1090, Austria; 2 Department of Gynecologic Pathology, Medical University of Vienna, Vienna, Austria

**Keywords:** cervical cancer, lymph node density, prognosis

## Abstract

**Background:**

To evaluate the prognostic value of lymph node density (LND) in patients with lymph node-positive cervical cancer.

**Methods:**

A total of 88 consecutive patients were included in our study. Patients were treated with cisplatin-based concomitant chemoradiotherapy after surgical staging was performed at the Medical University of Vienna. Lymph node density, that is, the ratio of positive lymph nodes to the total number of lymph nodes removed, was assessed pathologically. Patients were stratified into two groups according to LND: patients with LND ⩽10% and patients with LND >10%. Lymph node density was correlated with clinicopathological parameters by *χ*^2^-tests. Univariate log-rank tests and multivariate Cox regression models were used to evaluate the association between LND and survival.

**Results:**

A significant correlation between LND and FIGO stage (*P*=0.03), but not patients' age (*P*=0.2), histological grade (*P*=0.8), and histological type (*P*=0.5), was observed. In a univariate survival analysis, LND (*P*=0.01; *P*=0.01), FIGO stage (*P*=0.01; *P*=0.008), and histological grade (*P*=0.03; *P*=0.04) were associated with disease-free and overall survival, respectively. Patients with LND >10% had impaired disease-free and overall survival rates compared with patients with LND ⩽10%. In a multivariate regression model, LND (*P*=0.01; *P*<0.05) and FIGO stage (*P*=0.002; *P*=0.002) were independent predictors of disease-free and overall survival, respectively.

**Conclusions:**

LND >10% is associated with an impaired disease-free and overall survival. Lymph node density may be used as an independent prognostic parameter in patients with lymph node-positive cervical cancer.

Lymph node involvement is the most important prognostic parameter for patients with cervical cancer (Committee on Practice Bulletins-Gynecology, 2002; Tseng *et al*, 2010). The presence of lymph node metastases significantly influences patient's outcome and therapeutic modalities more than any other clinical or pathological feature. Moreover, it has been shown that the number of involved lymph nodes has a proportionately adverse influence on the prognosis of patients who have undergone surgery (Tanaka *et al*, 1984). Patients with locally advanced cervical cancer and lymph node involvement are best treated with primary radiotherapy (external beam plus brachytherapy) and concomitant chemotherapy. It has been shown that concomitant chemotherapy improves 5-year survival rates by 30–50 percent compared with radiotherapy alone (Committee on Practice Bulletins-Gynecology, 2002; Green *et al*, 2005).

Several recent studies have focused on the clinical relevance of lymph node density (LND) in assessing cancer prognosis (Herr, 2003; Chan *et al*, 2007; Ooki *et al*, 2007; Kassouf *et al*, 2008; Yamashita *et al*, 2008; Cai *et al*, 2009; Gil *et al*, 2009; Svatek *et al*, 2009; Vinh-Hung *et al*, 2009). In these studies, LND was defined as the ratio of the number of metastatic lymph nodes and the total number of lymph nodes removed. Thus, this parameter incorporates not only the burden of nodal disease and cancer spread but also the extent of nodal dissection and surgical staging. To date, no study has assessed the prognostic value of LND in patients with cervical cancer.

The purpose of this study was to evaluate the role of LND in the prognosis of patients with lymph node-positive cervical cancer.

## Materials and methods

### Patients

Institutional review board approval for this study was obtained from the Ethics Committee of the Medical University of Vienna (IRB approval number: 246-2009). A total of 88 patients with invasive, nodal-positive cancer of the uterine cervix underwent systemic surgical lymph node staging and were enrolled in the present study. Patients were treated at the Department of Obstetrics and Gynecology, Division of Gynecologic Oncology, Medical University of Vienna, Austria between January 1995 and December 2008. Patients' clinical and survival data were extracted from an electronic database, retrospectively.

### Clinical management

Patients with invasive nodal-positive cervical cancer (FIGO IB1–IVA) were treated with chemoradiotherapy, after systemic surgical lymph node staging was performed, as described previously (Polterauer *et al*, 2008). Chemoradiotherapy was conducted, using weekly cisplatin (40 mg m^−2^) and concurrent external-beam radiation therapy to the whole pelvic area (with an aim of 80–85 Gy) with a four-field technique, followed up by intracavitary brachytherapy (50–55 Gy). Tissue samples obtained from staging operations were sent to the department of pathology for histological examination. Board-approved pathologists, specialised in gynaecological pathology, assessed the pathological specimens. Formalin-fixed samples from each defined topographical localisation were described macroscopically for size, consistency, and number of lymph nodes. Individual recognisable lymph nodes were divided and paraffin embedded. To guarantee detection of all the nodes, even small nodes, remaining fatty tissue was also embedded. Finally, paraffin blocks were serially sectioned and stained with haematoxylin and eosin for microscopical examination. The number and location of total resected and metastatically involved pelvic and paraaortic lymph nodes was assessed.

After completion of concurrent chemoradiotherapy, patients were scheduled for the first follow-up visit 3 months after completion of therapy. For the next 3 years, patients were followed up every 3 months, in the fourth and fifth year bi-annually, and yearly from the sixth to the tenth year after primary therapy. If patients did not present at scheduled follow-up visits, they were contacted by administrative personnel. If any clinically suspicious finding and/or tumour marker elevation was detected, computed tomography was performed. Following international guidelines, recurrent disease was either diagnosed clinically, by biopsy, or by imaging methods. Patient with recurrent disease were either treated with single- or multidrug chemotherapy or with surgery. Documentation of death and causes of death was performed using post-mortem inspection or autopsy results.

### Statistical analysis

Values are given as mean (standard deviation) or median (inter-quartile range). The *χ*^2^-tests were used to compare LND and clinicopathological parameters. Patients were stratified into two risk groups according to LND (<10% *vs* ⩾10%). The cutoff value was chosen based on preliminary data (Ooki *et al*, 2007). Lymph node density was correlated with FIGO stage (I *vs* II *vs* III *vs* IV), patients' age (<50 *vs* ⩾50 years), histological grade (G1 *vs* G2 *vs* G3), and histological type (squamous cell *vs* adeno- and adenosquamous carcinoma).

Univariate survival probabilities were calculated by the product limit method of Kaplan and Meier. Differences between groups were tested using the log-rank test. Multivariate Cox proportional hazard models for disease-free and overall survival were performed. The results were analysed for the end point of disease-free and overall survival. Survival time periods of patients who are disease-free or still alive or dead as a result of other causes were censored with the last follow-up date. Univariate sub-group analyses according to FIGO stage were performed subsequently. The *P*-values of <0.05 were considered statistically significant. We used the statistical software SPSS 16.0 for Windows (SPSS 16.0, SPSS Inc, Chicago, IL, USA) for statistical analysis.

## Results

Mean age of patients at diagnosis was 49. 9 (14.1) years. Of the 88 patients, 86 (98%) and 2 (2%) patients had pelvic and paraaortic lymph node involvement, respectively. A mean/median number of 20.3 (11.6)/18.5 (12–27) and 3.1 (3.8)/2.0 (1–3) lymph nodes was resected or involved, respectively. A total of 36 (41%) and 52 (59%) patients were assigned to the LND group ⩽10% and >10%, respectively. Mean/median follow-up time for this cohort of patients was 37.1 (28.0)/34.5 (14–57) months. Patients' characteristics are given in Table 1.

In Table 2, we present the results of the association of LND and clinicopathological parameters. A significant association between LND groups and FIGO stage (*P*=0.03), but not patients' age (*P*=0.2), histological grade (*P*=0.8), and histological type (*P*=0.5), was observed.

**Figure 1:**
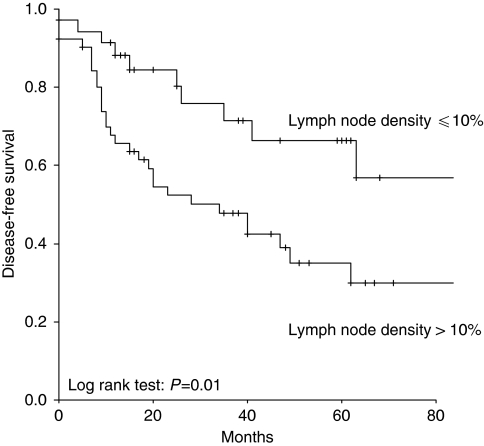
Kaplan–Meier curves for disease-free survival in patients with cervical cancer broken down by lymph node density.

**Figure 2:**
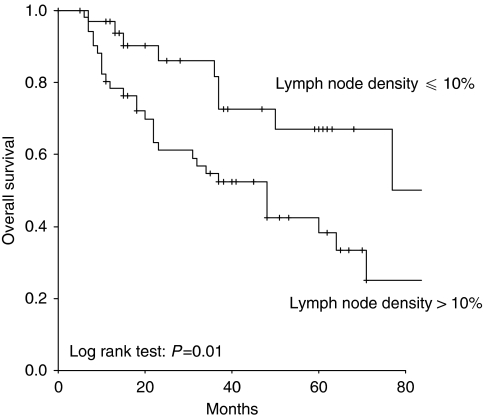
Kaplan–Meier curves for overall survival in patients with cervical cancer broken down by lymph node density.

In univariate survival analysis, LND (*P*=0.01; *P*=0.01), FIGO stage (*P*=0.01; *P*=0.008), and histological grade (*P*=0.03; *P*=0.04) were associated with disease-free and overall survival. Kaplan–Meier curves according to LND groups and disease-free and overall survival are shown in Figures 1 and 2, respectively. Patients of the LND ⩽10% group compared with the LND >10% group had 5-year overall survival rates of 67 and 38%, respectively. In multivariate survival analysis, only LND (*P*=0.01; *P*<0.05) and FIGO stage (*P*=0.002; *P*=0.002) were associated with disease-free and overall survival, respectively. Patients with LND >10% showed impaired disease-free and overall survival compared with patients with LND ⩽10%. Results of the univariate and multivariate survival analyses are provided in Table 3. A univariate sub-group survival analysis according to FIGO stages showed that LND was associated with overall survival in patients with FIGO stage I (*P*=0.01), but not with stage II (*P*=0.7), III (*P*=0.8), and IV (*P*=0.3). In a multivariate analysis, LND was not associated with survival in patients with FIGO stage I (*P*=0.9).

## Discussion

We investigated the prognostic value of LND for survival in patients with lymph node-positive cancer of the uterine cervix. We observed a strong association between LND and disease-free and overall survival.

Nodal metastases at the time of initial evaluation in patients with cervical cancer portend an impaired prognosis. The 5-year survival rates of women with stage IB cervical cancer with and without lymph node metastasis are 73.1 and 87.7%, respectively. Women with stage IIA cervical cancer with and without lymph node metastasis have 5-year survival rates of 40.9 and 79.8%, respectively (Lee *et al*, 1989). Owing to its powerful predictive ability, the incorporation of nodal status into clinical cervical staging has been discussed (Committee on Practice Bulletins-Gynecology, 2002). A number of studies have observed that location and extent of positive lymph nodes are important determinants in patients with cervical cancer (Tanaka *et al*, 1984; Lai *et al*, 2003; Singh and Arif, 2004). Decreased disease-free and overall survival rates have been reported in patients with an increasing number of positive lymph nodes and in patients with paraaortic lymph node metastases (Delgado *et al*, 1990).

We observed that patients with a higher LND had impaired disease-free and overall survival rates. In multivariate survival analyses, only FIGO stage and LND remained independent predictors of survival. A considerable number of our patients (36/88 (41%)) had LND ⩽10%. Of note, the 5-year overall survival rates of these patients are comparable with patients with FIGO stage IIA without lymph node involvement (Singh *et al*, 2004). These findings are novel and may assist the physician to better define prognosis and more importantly, stratify patients into various risk groups in the design of future clinical trials. Furthermore, our results can be helpful for patient counselling as patients with LND ⩽10% seem to have similar prognosis as patients with locally advanced disease without lymph node metastases. One might argue that the good prognosis in LND ⩽10% could be caused by the low number of positive lymph nodes in this group. To show prognostic superiority of LND to the number of positive lymph nodes, we performed a survival analysis regarding the number of positive lymph nodes (1 *vs* 2 *vs* >2). In our data set, this parameter was not significantly associated with overall survival (*P*=0.08).

Sub-group analysis was performed to characterise the additional prognostic benefit of LND in patients within the same tumour stage. Lymph node density >10% was associated with impaired overall survival in a univariate sub-group analysis for patients with FIGO stage I. In a multivariate model, we could not confirm these findings. This might be related to the relatively small number of patients (*n*=30) and deaths (*n*=6) in this sub-group.

In our study group, the median number of resected nodes was relatively high (18.5 (12–27)). According to the Gynecologic Oncology Group Surgical Procedures Manual (revised in 2005, www.gog.org), an adequate lymph node dissection requires that a minimum of four lymph nodes be shown pathologically from each side (right and left) of the pelvis, preferably from multiple sites.

Our results are in accordance with findings in other malignancies. A prognostic value of LND has been shown for several solid tumours, such as endometrial, breast, bladder, renal, prostate, oesophageal, gastric, oral, and penile cancer (Herr, 2003; Chan *et al*, 2007; Ooki *et al*, 2007; Kassouf *et al*, 2008; Yamashita *et al*, 2008; Cai *et al*, 2009; Gil *et al*, 2009; Svatek *et al*, 2009; Vinh-Hung *et al*, 2009). In patients with renal cancer, LND has been shown to be a more important predictor for prognosis than tumour stage or lymph node involvement (Kassouf *et al*, 2008). Lymph node density has been shown to provide more accurate prognostic information than the total number of positive nodes (Vinh-Hung *et al*, 2009), and its incorporation in a novel staging system has been suggested. A change in surgical staging would have clinical implications on treatment planning and follow-up.

Of note, a shortcoming of our study is the limited number of patients and the retrospective design. Furthermore, the treatment period of 13 years is relatively long.

We showed that LND is an independent prognostic parameter in patients with lymph node-positive cervical cancer and that it can be considered as a useful clinical tool, allowing classification into prognostic sub-groups. We believe that LND adds important information not only related to disease burden but also to staging quality. Lymph node density is influenced by surgical technique, anatomic circumstances and the quality and accuracy of the pathological analysis, reflecting an objective parameter in the assessment of the procedure's extent and completeness. For the subset of patients with favourable prognosis (that is, LND ⩽10%) regular follow-up may be a suitable choice, whereas patients with an unfavourable prognosis (that is, LND >10%) might possibly benefit from consolidation chemotherapy or another novel adjuvant treatment modality after chemoradiotherapy. However, these novel findings need to be validated in a larger, prospective, independent data set.

## Figures and Tables

**Table 1:** Patients' characteristics

**Parameter**	***N* or mean (s.d.)**
Total number of patients enrolled	88
Age at diagnosis (years)	49.9 (14.1)
	
*Histological type*	
Squamous cell carcinoma	71 (80.7%)
Adenocarcinoma	12 (13.6%)
Adenosquamous carcinoma	5 (5.7%)
	
*Tumour stage*	
FIGO IB1	20 (22.7%)
FIGO IB2	10 (11.4%)
FIGO IIA	7 (8.0%)
FIGO IIB	38 (43.2%)
FIGO IIIA	2 (2.3%)
FIGO IIIB	9 (10.2%)
FIGO IVA	2 (2.2%)
	
*Parametrial involvement*	
Negative	39 (44.3%)
Positive	49 (55.7%)
	
*Histological grade*	
G1	7 (8.0%)
G2	49 (55.7%)
G3	32 (36.3%)
	
*Recurrence status*	
No. of patients with recurrent disease	40 (45.5%)
Mean time to recurrent disease (months)	18.9 (16.9)
	
*Status at last observation*	
Alive with no evidence of disease or stable disease	46 (52.3%)
Progressive disease	4 (4.5%)
Tumour-related death	34 (38.6%)
Dead as a result of other causes	4 (4.5%)

Abbreviations: FIGO=International Federation of Gynecologists and Obstetricians; s.d.=standard deviation.

**Table 2:** Relationship between clinicopathological parameters and lymph node density in 88 patients with lymph node-positive cervical cancer

	**LND ⩽10% (*n*=36)**	**LND >10% (*n*=52)**	***P*-value** a
*Tumour stage*			0.03
FIGO I	18/30 (60.0%)	12/30 (40.0%)	
FIGO II	13/45 (28.8%)	32/45 (71.2%)	
FIGO III and IV	5/13 (38.5%)	8/13 (31.5%)	
			
*Age at first diagnosis*			0.2
<50 years	22/48 (45.8%)	26/48 (54.2%)	
⩾50 years	14/40 (35.0%)	36/52 (65.0%)	
			
*Histological grade*			0.8
G1	2/6 (33.3%)	4/6 (66.7%)	
G2	20/46 (43.5%)	26/46 (56.5%)	
G3	11/30 (36.7%)	19/30 (63.3%)	
			
*Histological type*			0.5
Squamous cell carcinoma	28/71 (39.4%)	43/71 (60.6%)	
Adenosquamous carcinoma	7/16 (43.8%)	9/16 (56.2%)	

Abbreviations: FIGO=International Federation of Gynecologists and Obstetricians; LND=lymph node density.

a *χ*^2^-test.

**Table 3:** Univariate and multivariate survival analyses in 88 patients with lymph node-positive cervical cancer

	**Disease-free survival**			**Overall survival**		
	**Univariate** a	**Multivariate** b		**Univariate** a	**Multivariate** b	
	***P*-value**	***P*-value**	**HR (95% CI)**	***P*-value**	***P*-value**	**HR (95% CI)**
Lymph node-density (⩽10% *vs* >10%)	0.01	0.03	2.2 (1.1–4.7)	0.01	0.046	2.2 (1.0–4.8)
FIGO stage (I *vs* II *vs* III *vs* IV)	0.01	0.002	2.0 (1.3–3.2)	0.008	0.002	2.1 (1.3–3.4)
Patients' age	0.5	0.07	1.0 (0.9–1.1)	0.8	0.09	0.9 (0.9–1.0)
Histological grade (G1 *vs* G2 *vs* G3)	0.03	0.09	1.6 (0.9–2.7)	0.05	0.07	1.7 (0.9–2.9)
Histological type (squamous cell carcinoma *vs* adenosquamous carcinoma)	0.01	0.03	2.2 (1.1–4.7)	0.2	0.08	2.0 (0.9–4.4)

Abbreviations: 95% CI=95% confidence interval; FIGO=International Federation of Gynecologists and Obstetricians; HR=hazard ratio.

a Log-rank test.

b Multivariate Cox-regression analysis.
